# Stroke prevalence among the Spanish elderly: an analysis based on screening surveys

**DOI:** 10.1186/1471-2377-6-36

**Published:** 2006-10-16

**Authors:** Raquel Boix, José Luis del Barrio, Pedro Saz, Ramón Reñé, José María Manubens, Antonio Lobo, Jordi Gascón, Ana de Arce, Jaime Díaz-Guzmán, Alberto Bergareche, Félix Bermejo-Pareja, Jesús de Pedro-Cuesta

**Affiliations:** 1 National Centre for Epidemiology, Carlos III Institute of Public Health, Madrid, Spain; 2 Department of Medicine and Psychiatry, Zaragoza University, Zaragoza, Spain; 3 Dementia Diagnosis and Treatment Unit, Neurology Department, Bellvitge University Teaching Hospital, Barcelona, Spain; 4 Neurology Unit, Virgen del Camino Hospital, Pamplona, Spain; 5 Neurology Department, Donostia Hospital, Guipúzcoa, Spain; 6 Neurology Department, 12 de Octubre University Teaching Hospital, Madrid, Spain; 7 Neurology Department, Bidasoa-Hondarribia Hospital, Guipúzcoa, Spain

## Abstract

**Background:**

This study sought to describe stroke prevalence in Spanish elderly populations and compare it against that of other European countries.

**Methods:**

We identified screening surveys -both published and unpublished- in Spanish populations, which fulfilled specific quality requirements and targeted prevalence of stroke in populations aged 70 years and over. Surveys covering seven geographically different populations with prevalence years in the period 1991–2002 were selected, and the respective authors were then asked to provide descriptions of the methodology and raw age-specific data by completing a questionnaire. In addition, five reported screening surveys in European populations furnished useful data for comparison purposes. Prevalence data were combined, using direct adjustment and logistic regression.

**Results:**

The overall study population, resident in central and north-eastern Spain, totalled 10,647 persons and yielded 715 cases. Age-adjusted prevalences, using the European standard population, were 7.3% for men, 5.6% for women, and 6.4% for both sexes. Prevalence was significantly lower in women, OR 0.79 95% CI 0.68–0.93, increased with age, particularly among women, and displayed a threefold spatial variation with statistically significant differences. Prevalences were highest, 8.7%, in suburban, and lowest, 3.8%, in rural populations. Compared to pooled Spanish populations, statistically significant differences were seen in eight Italian populations, OR 1.39 95%CI (1.18–1.64), and in Kungsholmen, Sweden, OR 0.40 95%CI (0.27–0.58).

**Conclusion:**

Prevalence in central and north-eastern Spain is higher in males and in suburban areas, and displays a threefold geographic variation, with women constituting the majority of elderly stroke sufferers. Compared to reported European data, stroke prevalence in Spain can be said to be medium and presents similar age- and sex-specific traits.

## Background

It is accepted that stroke constitutes the second leading cause of death and leading determinant of disability among the world adult population [1] and, moreover, that its health impact will increase in the future [2]. Mortality due to cerebrovascular disease (CVD) and other disorders in Spain is monitored by the National Centre for Epidemiology [3]. These data show that the leading cause of death among women and the third leading cause of death, after ischaemic heart disease and lung cancer, among men, is cerebrovascular disease, with the highest rates being registered in the southern half of the country following a well-documented time trend [4]. CVD mortality per 100,000 population levelled off in the period 1992–2002, declining from 87.04 to 61.25 among men and from 80.09 to 48.12 among women. From different hospital-based reports, it would appear that 80% of CVD in the Spanish adult population is due to ischaemic lesions and approximately 20% to either parenchymal or subarachnoidal brain haemorrhage [5]. Studies on cost of stroke care report different results [6,7]. Yet it seems that stroke-unit development and the timing and distribution of rehabilitation for stroke patients in Spain differ from that seen in other European populations, with the use of such resources being sparse and largely allocated to young, severely affected patients [8-11].

Lack of data and focus on different entities (first-ever, minor stroke, etc.) and population age-strata have meant that incidence of stroke in Spanish populations is not well known [12]. To date, the most reliable data on stroke frequency in Spain have been in the form of prevalences yielded by door-to-door surveys, which were conducted in the period 1990–2000 on five Spanish populations aged 70 years and over, and reported figures of 4.6% to 11.5% for men and 5.2% to 7.9% for women [13]. Nevertheless, this report failed to encompass unpublished results from other door-to-door surveys conducted in Spain. Our study adds data on five new study populations and 247 new stroke cases. Since a reported review of stroke prevalence, based on screening surveys in European populations including two Spanish surveys [14], failed to reveal relevant differences, comparable European and pooled Spanish data were combined in this study using logistic models.

Accordingly, our study sought to: update data on stroke prevalence in Spanish elderly populations, by including results from new surveys and sub-populations; and compare new age- and sex-specific prevalence counts, using models and references from reported European door-to-door surveys.

## Methods

In this report, we followed the recommendations of the Meta-analysis of Observational Studies in Epidemiology (MOOSE) Group [15]. A search strategy for identification of screening surveys was implemented in September 2005 by a librarian, using PubMed, Indice Médico Español (IME) and Biblioteca Virtual en Salud (BVS-Bireme), and the key words, "stroke", "prevalence", "door-to-door", and "Europe". The restrictions imposed were as follows: English or Spanish language; and publication year 1985 to 2005. The search yielded 303 reports in English and 14 in Spanish. In addition, Spanish authors of unreported surveys were contacted personally by researchers or by scientific societies. These strategies provided data on 10 stroke prevalence surveys conducted in Spanish populations and eight in European populations, using a screening approach.

In a second step, we identified screening surveys targeting prevalence of stroke in Spanish populations, which fulfilled the following quality criteria, explicitly mentioning: 1) use of an updated population census for a study population geographically defined by residence; 2) use of a screening instrument in the first phase of the study; 3) a description of the clinical work-up and type of medical specialist responsible for diagnostic ascertainment in phase II; and, 4) use of defined diagnostic criteria or requirements for classification in stroke diagnostic categories for assignment of a specific prevalence numerator. A number of such studies conducted in central and northern Spain were identified, including a pilot study [16], and five surveys covering seven geographically different populations [14,17-19], namely, Zaragoza, Pamplona, Lista, Las Margaritas, Arévalo and Gerona. Three other unpublished screening surveys conducted in populations in the Basque Country [20], El Prat in Catalonia, and Alcoi/Bañeres [21] in south-east Spain were identified from contacts provided by researchers. The studies in Gerona [19] and Alcoi/Bañeres [21] were excluded from our study owing to difficulties in access to data or incomplete data reported by authors. Since some studies solely covered populations aged seventy years and over, the subpopulations meeting the requirements for inclusion in the re-analysis of selected studies comprised subjects over the age of 69 years, divided into ten, 5-year age- and sex-specific strata.

A panel of experts -RB, JLB and JPC- designed a questionnaire for data-collection on the basis of different studies focusing on demographic, methodological, diagnostic, disability and epidemiological data, and resolved issues concerning the diagnostic classification of specific individuals.

The ill health of co-author, JMM, rendered data-collection in Pamplona unfeasible, and reported data were thus used instead.

Population and methodological characteristics of the selected studies [22-25] are listed in Table 1. Prevalence years for Spanish and non-Spanish studies were different, ranging from 1991 to 2002 and 1987 to 2001 respectively The population ranged from 1,010 in Lista to 2,850 in Zaragoza, and the number of cases went from 47 in Lista and Las Margaritas to 208 in El Prat de Llobregat. The information was recorded in a database listing age-and sex-specific groups, study, population and cases, for each survey. Demographic characteristics relating to the population concentration categories of municipal populations, which generally proved to be larger than those surveyed, were obtained from the National Institute of Statistics [26]. Rural populations had under 2,000 and urban populations over 10,000 inhabitants. Urban metropolitan populations with a high proportion of immigrants were denoted as suburban. An urban mixed category was used to identify former rural populations which had become urban in recent decades.

In addition to descriptive statistics, e.g., prevalence proportions, age- and sex-specific, as well as crude and age-adjusted using the European standard population [27] (its age distribution being similar to those of the study populations), the statistical analysis also included comparisons using unconditional logistic regression on grouped data, Stata version 8.0, with the population of El Prat de Llobregat taken as reference, due to its large size and recent prevalence date. The dependent variable was cases and the independent variables were sex, study and age group.

For the purposes of comparison with stroke prevalence in European studies, we selected five of the eight identified door-to-door stroke surveys in European populations (Rotterdam, Kungsholmen, ILSA, SNES and Vecchiano) [14,28,29] that fulfilled the quality criteria applied to Spanish surveys, and rejected those conducted in Patras, the north-west Peloponnese and Rome [30-32], since the age- and sex-specific groups used were too wide, (i.e., 10 years). In addition to visual comparison from graphs, logistic regression was used taking the pooled Spanish populations as reference. Different models were used for comparisons, determined by age-intervals available from surveys in different European populations.

## Results

**Figure 1:**
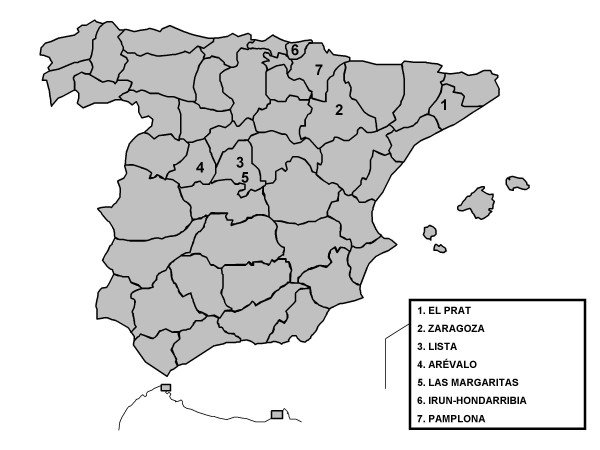
Geographic location of surveys.

The geographical distribution of the study populations is depicted in Figure 1. Surveys were located in the central and north-eastern regions of mainland Spain. In terms of habitat, the Zaragoza, Lista and Pamplona surveys focused on urban populations, those in Margaritas and El Prat were suburban, with a high municipal proportion of immigrant populations (i.e., born outside the municipal boundaries), 36% and 33% respectively, while others were less homogeneous, with Arévalo classified as rural and the Bidasoa Region, made up of two larger, partly industrial and rural municipalities having a 25% immigrant population, designated as urban-mixed.

**Figure 2:**
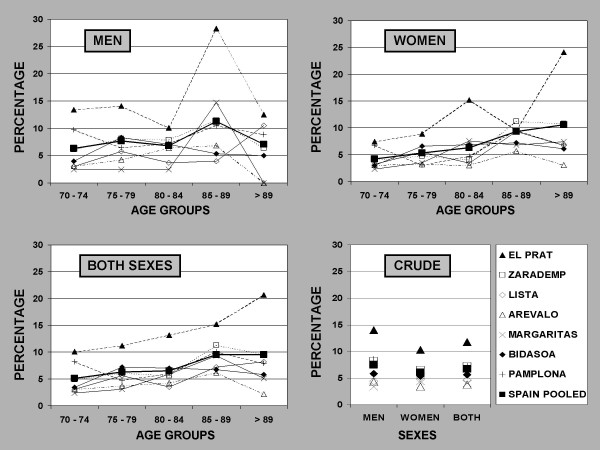
Crude and age-specific prevalence of stroke in Spanish populations.

The overall analysis was based on a study population of 10,647, comprising 4,400 (41%) men and 6,247 (59%) women, and 715 cases, 331 male and 384 female. Detailed age- and sex-specific prevalences are shown in Figure 2 and Table 2, in which crude and age-adjusted prevalences are also provided. The age- and sex-specific patterns suggested an increase in prevalence with age among women and a decrease in prevalence among the oldest men aged 90 years and over. As can be seen in Figure 2, there was a considerable – approximately threefold- variation in age-specific prevalences reported by the different surveys, with age-adjusted values ranging from 3.8% in Arévalo to 11.9% in El Prat. Crude overall prevalences for ages 70 and over were 7.5% among men and 6.1% among women and 6.7% for both sexes, with corresponding age-adjusted values of 7.3%, 5.6% and 6.4% respectively. The logistic regression results summarised in Table 3 show prevalences as being significantly, lower in women OR 0.79 95%CI 0.68–0.93, increasing with age, and registering a considerable -approximately twofold- spatial variation, with significant differences between El Prat and five other geographical areas. The sex-specific pattern was consistent with clearer, statistically significant increases with age among women.

**Figure 3:**
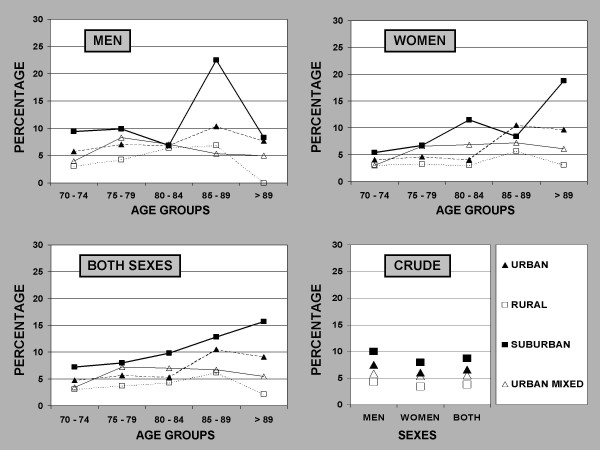
Crude and age-specific prevalence of stroke in Spanish populations grouped by habitat.

Habitat-specific prevalences, broken down by gender and age category, are presented in Figure 3 and Table 4, with the highest and lowest age-adjusted proportions being registered for the suburban and rural populations, 8.7% and 3.8% respectively. Logistic regression showed that, as against urban populations, the differences proved statistically significant for both suburban OR 1.50 95% CI 1.26–1.79 and rural areas OR 0.64 95% CI 0.48–0.86.

**Figure 4:**
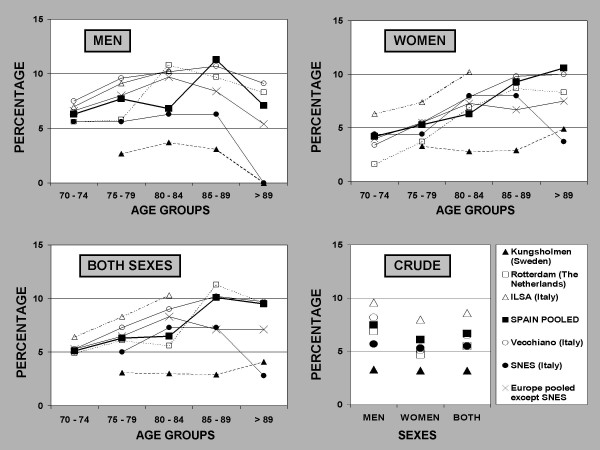
Crude and age-specific prevalence of stroke in pooled Spanish and in European populations.

European comparisons were deemed to be valid vis-à-vis Rotterdam (The Netherlands), Vecchiano (Italy) and Kungsholmen (Sweden) for age intervals 75 and over, the Italian ILSA survey for ages 70–85 years, the SNES study (Sicily-Italy) for ages 70 years and over, but not vis-à-vis the two Greek surveys where the population aged 70 and over was collapsed into just one group. The age-specific pattern (see Figure 4) suggests that prevalences in Spanish populations were lower than those in the Italian ILSA survey and Vecchiano, and higher than those in Rotterdam, Sicily and Kungsholmen. Logistic models, Table 5, showed that, when prevalence in pooled Spanish populations was taken as reference, such differences proved to be statistically significantly higher in ILSA, OR 1.39 95% CI 1.18–1.64, and lower in Kungsholmen, OR 0.40 95% CI 0.27–0.58. Internationally, women also had a lower risk ranking, OR 0.78 95% CI 0.67–0.91 to OR 0.81 95% CI 0.70–0.94, in the above models.

Among the prevalent clinical population with stroke, the proportion of women rose from 46% at ages 70–74 years to 54% at age 90 years and over, while at age 80 years and over this same proportion was 60%.

## Discussion

According to our estimates, prevalence of stroke in central and north-eastern Spain is higher in males and in suburban areas, and displays a threefold geographic variation. Compared to reported European data, stroke prevalence in Spain is medium. Prevalence appears to increase with age, particularly among women, and falls sharply at ages 90 and over, particularly among men, a pattern shared with the European population. Women account for the majority of the prevalent clinical population with stroke.

This study constitutes the first overview of stroke prevalence in Spanish elderly populations. The fact that it included both published and unpublished studies might have served to control for any possible publication bias, though it has to be said that only reported studies were included in the comparison with European data. Furthermore, the inclusion of unpublished studies might shed light on spatial variation of stroke occurrence in Spain where mortality due to stroke varies remarkably. Unfortunately, stroke prevalence figures for Spanish and European elderly populations obtained from door-to-door surveys are scarce. Since most of the Spanish surveys excluded were unpublished and the quality of the data was low, we feel that restriction to English in the literature search is unlikely to have left a substantial number of quality surveys undetected. Insofar as type of stroke was concerned, prevalence data were to all intents and purposes non-existent.

Although an international comparison of stroke prevalence in Spanish populations has been reported [14], only the Pamplona and Zaragoza studies were included and pooled with the southern European population in comparisons. Differences between our results and those from a prior review of reported stroke prevalence in Spain [13] reside in the data for a small rural population in the Arévalo municipal area, for which the latter pilot study registered a high prevalence.

Interpretation of the results from our intra- and inter-country comparisons is limited by a number of factors. In the case of the Spanish surveys, some methodological differences were in evidence, namely: a formal screening instrument was used in the Bidasoa [33] and NEDICES [34] studies; and training and health-professional profiles of field workers varied. We believe that such methodological differences may have determined differences in prevalences. However, this interpretation would be difficult to reconcile with a similar sex-specific, spatial mortality pattern reported for the period 1985–1998, with lowest rates in The Netherlands and Sweden, medium rates in Spain and highest rates in Italy [4]. Unfortunately, a hypothetical role postulated for the incidence underlying these two patterns would be difficult to verify due to the lack of comparable stroke-incidence figures in the literature.

A remarkable characteristic of the geographical variation in prevalence in Spanish populations are the high discordant figures registered for El Prat, with most of the remaining values lying in the medium to low ranges. Frequencies of vascular risk factors in Spain described by the National Health Survey and a recent meta-analysis [35,36] might not be useful for inference at such small-size populations. However, a tantalizing interpretation of regional differences in stroke prevalence here found might be to attribute them to variation of prevalence of vascular risk factors, in view of the fact that from a recent review of prevalence of vascular risk factors in Spanish populations door-to-door screened for neurological disease [37] a systematic, while not statistically significant, higher prevalence of diabetes, hypertension, tobacco use and hypercholesterolaemia were found between El Prat and Arévalo. The fact that El Prat survey was the most recently conducted, that screening was performed by a neurologist, and that a considerable part of the immigrant population came from Andalusia and Extremadura -both being southern Spanish regions where stroke mortality has been reported to be highest [38] – we are inclined to speculate that the high prevalence found in El Prat might in part be explained by high stroke ascertainment in a population at high risk for stroke. Since the abovementioned differences in frequency of risk factors were modest, the lowest prevalence found in rural populations (Arévalo) based on 53 cases might, however, be more difficult to attribute to specific determinants, not being able to exclude underreporting.

Important age- and sex-related patterns of stroke prevalence, such as the fall in prevalence among elderly men, the rising trend among women, and the high percentage of women in the elderly stroke population are shared by the Spanish and remaining European populations. These traits may characterise stroke in Spain possibly suggesting: 1) that women develop stroke at a later age than do men, as observed in Spanish studies [14,39]; 2) that male stroke sufferers have worse survival prospects than do their female counterparts; and 3) that such sex-selective survival is particularly evident at very old ages. Since the latter two statements have not been empirically supported by the results of European studies, in which age-adjusted case-fatality rates were higher among women than among men [40,41], the above-mentioned pattern in Spain might be explained by the effect of differential incidence traits in the two sexes. However, the pattern in some European populations appears to be more difficult to be explained by differences in incidence, namely because incidence traits between sexes have not be found to be differential but identically rising, for example in the screening survey on first-ever stroke in the Rotterdam cohort [14].

Questions to be answered by future research may well refer to stroke prevalence in western and southern Spain, prevalence of stroke by type, and detailed descriptions of the clinical-population numerators. Stroke prevalence surveys should incorporate the study of stroke sufferers as well as the view held by the population of care of such patients. The increasingly advanced ageing of the Spanish population suggests that the burden of stroke in Spain will increase, thus calling for specific research.

## Conclusion

To reiterate, we describe stroke prevalence in central and north-eastern Spain, which, compared to reported European data, is medium. Furthermore, prevalence in these regions of Spain is higher in males and in suburban areas, and displays a threefold geographic variation, with women constituting the majority of elderly stroke sufferers.

## Competing interests

The author(s) declare that they have no competing interests.

## Authors' contributions

RB took part in designing the reanalysis methods and drafted the first manuscript. JLB participated in design and co-ordination, performed the statistical analysis and contributed to drafting the manuscript. PS, RR, AL, JG, ADA, JDG, JMM, AB and FBP collaborated on study design and data collection, and PS, RR, AL, JG, ADA, JDG, AB and FBP participated in a critical review of the manuscript. JPC (the study co-ordinator) and FBP conceived the study, participated in the overall design and helped lend the paper its final shape. All authors have read and approved the final manuscript.

## Pre-publication history

The pre-publication history for this paper can be accessed here:



## Figures and Tables

**Table 1:** Methodological aspects of selected, door-to-door stroke prevalence surveys

**Survey acronym (country)**	**Study population's residence**	**Type of sample**	**Number of individuals studied**	**Number of cases**	**Age (Years)**	**Prevalence year**	**Percentage collaboration at screening**	**Screening phase Instrument & Field workers**	**Diagnostic ascertainment phase**	**Diagnostic criteria for stroke**
PRATICON (Spain)	El Prat de Llobregat (Barcelona)	Random sample of municipal voters roll	1,754	208	≥ 70	2002	85	Questionnaire Possible stroke symptoms. Field workers: trained neuropsychologist	Stroke protocol. Neurological examination. Hachinski ischaemic score. Brain imaging.	Ischaemic stroke and TIA separately
ZARADEMP (Spain)	Zaragoza municipal area	Random sample of municipal voters roll	2,850	205	≥ 55	1996	83	Questionnaire Possible stroke symptoms. Field workers: trained medical students	Consensus based on review of study data.	Ischaemic stroke and TIA separately Confirmed and possible
BIDASOA (Spain)	Irún, Hondarribia (Guipúzcoa)	Random sample of municipal voters roll	1,349	75	≥ 65	1996	85	Questionnaire Clinical examination SNES (Sicilian Neuro- Epidemiological Study) Field workers: trained sociology students	Neurological examination. Review of medical records.	Thrombotic ischaemic stroke Embolic ischaemic stroke Parenchymatous haemorrhagic stroke Subarachnoid haemorrhage Unspecified stroke Confirmed/possible
PAMPLONA (Spain)	Pamplona (Navarre)	Random sample of municipal voters roll	1,127	80	≥ 70	1991	78	Questionnaire Clinical examination. Field workers: trained physicians	Consensus based on review of study data.	WHO indications [22]
NEDICES (Spain)	Lista (Madrid) Las Margaritas (Getafe, Madrid) Arévalo (Avila)	Random sample of municipal voters roll	1,010 1,164 1,393	47 47 53	≥ 65	1994	78 89 95	Questionnaire. Monica Project items. Review of medical records. Field workers: trained physicians	Neurological examination. Review of medical records.	WHO indications [22]
KUNGSHOLMEN (Sweden)	Kungsholmen, Stockholm	Census	984	31	≥ 75	1987	77	No screening phase.	Self-reported diagnosis. Review of medical records. Clinical examination of the whole population.	Consensus based on review of study data.
ROTTERDAM (The Netherlands)	Rotterdam district	Sample of general population	2,347	158	≥ 55	1993	78	Questionnaire. Incidence. Regular reports of automatic GP records. Field workers: not specified	Review of medical records and study data. Diagnosis by neurologist or GP.	Medically diagnosed stroke. Symptoms more than 24 hours
ILSA (Italy)	Genoa, Segrate (Milan), Selvazzo- Rubano (Padua), Catania, Impruneta (Florence), Fermo (Ascoli Piceno), Naples and Casamassima (Bari).	Random sample of municipal voters roll	3,343	280	≥ 65	1992	80	Questionnaire Possible stroke symptoms. Simple neurological examination. Field workers: not specified	Neurological examination. Review of medical records. Consensus diagnosis.	WHO indications [23] Oxfordshire Community stroke project [24]
SNES (Italy)	Riposto, Santa Teresa di Riva and Terrasini (Sicily)	All subjects residing in municipalities	2,094	115	≥ 65	1987	92	Clinical examination SNES (Sicilian Neuro- Epidemiological Study) Field workers: medically trained	Neurological examination. Review of medical records. Consensus diagnosis.	Modified Schoenberg et al criteria [25]
Vecchiano (Italy)	Vecchiano, North-west Tuscany	All inhabitants aged 65 years or over	1,136	96	≥ 65	2001	95	Clinical examination. Field workers: investigator trained in the diagnosis of cerebrovascular diseases	Neurological examination.	Symptom questionnaire with six questions

**Table 2:** Age- and sex-specific prevalence of stroke in different surveys.

**MEN**							
**LOCATION (STUDY)**	**Age Group**					**Crude** cases/pop. P (%)	**Age-adjusted** P (%)
			
	**70 – 74** cases/pop*. P (%)**	**75 – 79** cases/pop. P (%)	**80 – 84** cases/pop. P (%)	**85 – 89** cases/pop. P (%)	**> 89** cases/pop. P (%)		

**1. El Prat de Llobregat (PRATICON)**	46/344 13.4	30/212 14.1	11/109 10.1	13/46 28.3	3/24 12.5	103/735 14.0	14.5
**2. Zaragoza (ZARADEMP)**	21/345 6.1	20/245 8.1	15/188 7.9	31/269 11.5	5/78 6.4	92/1.125 8.2	7.6
**3. Lista (NEDICES)**	4/135 3.0	7/121 5.8	3/82 3.7	2/50 4.0	2/19 10.5	18/407 4.4	4.3
**4. Arévalo (NEDICES)**	9/286 3.1	7/161 4.3	7/109 6.4	4/58 6.9	0/14 0.0	27/628 4.3	4.3
**5. Las Margaritas (NEDICES)**	5/198 2.5	3/120 2.5	2/80 2.5	5/34 14.7	0/12 0.0	15/444 3.4	3.7
**6. Hondarribia/Irún (BIDASOA)**	9/225 4.0	11/133 8.3	6/84 7.1	2/37 5.4	1/20 5.0	29/499 5.8	5.8
**7. Pamplona (PAMPLONA)**	7/71 9.8	10/152 6.5	11/152 7.2	15/142 10.5	4/45 8.9	47/562 8.4	8.6
**TOTAL**	101/1.604 6.3	88/1.144 7.7	55/804 6.8	72/636 11.3	15/212 7.1	331/4.400 7.5	7.3

**WOMEN**							

**LOCATION (STUDY)**	**Age Group**					**Crude** cases/pop. P (%)	**Age-adjusted** P (%)
			
	**70 – 74** cases/pop. P (%)	**75 – 79** cases/pop. P (%)	**80 – 84** cases/pop. P (%)	**85 – 89** cases/pop. P (%)	**> 89** cases/pop. P (%)		

**1. El Prat de Llobregat (PRATICON)**	30/405 7.4	25/280 8.9	26/171 15.2	10/105 9.5	14/58 24.1	105/1.019 10.3	10.0
**2. Zaragoza (ZARADEMP)**	20/492 4.0	18/372 4.8	12/292 4.1	41/366 11.2	22/203 10.8	113/1.725 6.5	5.3
**3. Lista (NEDICES)**	7/218 3.2	9/163 5.5	4/117 3.4	7/75 9.3	2/30 6.7	29/603 4.8	4.7
**4. Arévalo (NEDICES)**	9/290 3.1	6/187 3.2	5/168 3.0	5/88 5.7	1/32 3.1	26/765 3.4	3.4
**5. Las Margaritas (NEDICES)**	6/264 2.3	7/198 3.5	12/158 7.6	5/73 6.8	2/27 7.4	32/720 4.4	4.1
**6. Hondarribia/Irún (BIDASOA)**	9/302 3.0	15/228 6.6	12/174 6.9	7/97 7.2	3/49 6.1	46/850 5.4	5.1
**7. Pamplona (PAMPLONA)**	5/75 6.7	5/159 3.1	7/150 4.7	13/137 9.5	3/44 6.8	33/565 5.8	5.7
**TOTAL**	86/2.046 4.2	85/1.587 5.3	78/1.230 6.3	88/941 9.3	47/443 10.6	384/6.247 6.1	5.6

**BOTH SEXES**							

**LOCATION (STUDY)**	**Age Group**					**Crude** cases/pop. P (%)	**Age-adjusted** P (%)
			
	**70 – 74** cases/pop. P (%)	**75 – 79** cases/pop. P (%)	**80 – 84** cases/pop. P (%)	**85 – 89** cases/pop. P (%)	**> 89** cases/pop. P (%)		

**1. El Prat de Llobregat (PRATICON)**	76/749 10.1	55/492 11.2	37/280 13.2	23/151 15.2	17/82 20.7	208/1.754 11.8	11.9
**2. Zaragoza (ZARADEMP)**	41/837 4.9	38/617 6.1	27/480 5.6	72/635 11.3	27/281 9.6	205/2.850 7.2	6.2
**3. Lista (NEDICES)**	11/353 3.1	16/284 5.6	7/199 3.5	9/125 7.2	4/49 8.2	47/1.010 4.6	4.5
**4. Arévalo (NEDICES)**	18/576 3.1	13/348 3.7	12/277 4.3	9/146 6.2	1/46 2.2	53/1.393 3.8	3.8
**5. Las Margaritas (NEDICES)**	11/462 2.4	10/318 3.1	14/238 5.9	10/107 9.3	2/39 5.1	47/1.164 4.0	3.9
**6. Hondarribia/Irún (BIDASOA)**	18/527 3.4	26/361 7.2	18/258 7.0	9/134 6.7	4/69 5.8	75/1.349 5.6	5.4
**7. Pamplona (PAMPLONA)**	12/146 8.2	15/311 4.8	18/302 5.9	18/279 6.4	7/89 7.9	80/1.127 7.1	7.1
**TOTAL**	187/3.650 5.1	173/2.731 6.3	133/2.034 6.5	160/1.577 10.1	62/655 9.5	715/10.647 6.7	6.4

* pop. : Population

** P (%): Prevalence

**Table 3:** Prevalence OR with 95%CI from models, controlled for age and sex. Reference: EL Prat de Llobregat

	**BOTH SEXES OR (95%CI)**	**MEN OR (95%CI)**	**WOMEN OR (95%CI)**
**1. El Prat de Llobregat**	**1**	**1**	**1**
**2. ZARADEMP**	0.53 (0.43 – 0.65)	0.51 (0.38 – 0.70)	0.54 (0.41 – 0.72)
**3. Lista**	0.37 (0.27 – 0.52)	0.30 (0.18 – 0.50)	0.45 (0.30 – 0.70)
**4. Arévalo**	0.31 (0.23 – 0.43)	0.30 (0.19 – 0.47)	0.33 (0.21 – 0.51)
**5. Las Margaritas**	0.34 (0.24 – 0.47)	0.23 (0.14 – 0.41)	0.43 (0.29 – 0.65)
**6. Bidasoa**	0.46 (0.35 – 0.61)	0.41 (0.27 – 0.63)	0.51 (0.36 – 0.73)
**7. Pamplona**	0.49 (0.37 – 0.64)	0.51 (0.35 – 0.75)	0.47 (0.31 – 0.71)
**70 – 74**	**1**	**1**	**1**
**75 – 79**	1.26 (1.01 – 1.56)	1.23 (0.91 – 1.67)	1.29 (0.95 – 1.76)
**80 – 84**	1.36 (1.08 – 1.72)	1.15 (0.81 – 1.62)	1.59 (1.16 – 2.18)
**85 – 89**	2.10 (1.68 – 2.65)	1.87 (1.34 – 2.61)	2.34 (1.71 – 3.21)
**> 90**	1.91 (1.41 – 2.60)	1.13 (0.64 – 2.00)	2.54 (1.74 – 3.70)
**MEN**	**1**	-	-
**WOMEN**	0.79 (0.68 – 0.93)	-	-

**Table 4:** Age- and sex-specific prevalence of stroke by habitat.

**MEN**								
**HABITAT**	**Age Group**					**Crude** cases/pop. P (%)	**Age- adjusted** P (%)	**OR and 95%CI controlled for age and sex.** **Reference: URBAN**
				
	**70 – 74** cases/pop* P (%)**	**75 – 79** cases/pop. P (%)	**80 – 84** cases/pop. P (%)	**85 – 89** cases/pop. P (%)	**> 89** cases/pop. P (%)			

**1. URBAN**	32/551 5.8	37/518 7.1	29/422 6.9	48/461 10.4	11/142 7.7	157/2.094 7.5	6.9	**1**
**2. URBAN MIXED**	9/225 4.0	11/133 8.3	6/84 7.1	2/37 5.4	1/20 5.0	29/499 5.8	5.8	0.87 (0.57 – 1.31)
**3. SUBURBAN**	51/542 9.4	33/332 9.9	13/189 6.9	18/80 27.5	3/36 8.3	118/1.179 10.0	10.4	1.49 (1.15 – 1.94)
**4. RURAL**	9/286 3.1	7/161 4.3	7/109 6.4	4/58 6.9	0/14 0.0	27/628 4.3	4.3	0.63 (0.41 – 0.97)

**WOMEN**								

**HABITAT**	**Age Group**					**Crude** cases/pop. P (%)	**Age- adjusted** P (%)	**OR and 95%CI controlled for age and sex.** **Reference: URBAN**
				
	**70 – 74** cases/pop P (%)	**75 – 79** cases/pop. P (%)	**80 – 84** cases/pop. P (%)	**85 – 89** cases/pop. P (%)	**> 89** cases/pop. P (%)			

**1. URBAN**	32/785 4.1	32/694 4.6	23/559 4.1	61/578 10.5	27/277 9.7	175/2.893 6.0	5.1	**1**
**2. URBAN MIXED**	9/302 3.0	15/228 6.6	12/174 6.9	7/97 7.2	3/49 6.1	46/850 5.4	5.1	1.00 (0.71 – 1.40)
**3. SUBURBAN**	36/669 5.4	32/478 6.7	38/329 11.5	15/178 8.4	16/85 18.8	137/1.739 7.9	7.5	1.49 (1.18 – 1.89)
**4. RURAL**	9/290 3.1	6/187 3.2	5/168 3.0	5/88 5.7	1/32 3.1	26/765 3.4	3.4	0.64 (0.42 – 0.97)

**BOTH SEXES**								

**HABITAT**	**Age Group**					**Crude** cases/pop. P (%)	**Age- adjusted** P (%)	**OR and 95%CI controlled for age and sex.** **Reference: URBAN**
				
	**70 – 74** cases/pop P (%)	**75 – 79** cases/pop. P (%)	**80 – 84** cases/pop. P (%)	**85 – 89** cases/pop. P (%)	**> 89** cases/pop. P (%)			

**1. URBAN**	64/1.336 4.8	69/1.212 5.7	52/981 5.3	109/1.039 10.5	38/419 9.1	332/4.987 6.6	5.9	**1**
**2. URBAN MIXED**	18/527 3.4	26/361 7.2	18/258 7.0	9/134 6.7	4/69 5.8	75/1.349 5.5	5.4	0.95 (0.73 – 1.23)
**3. SUBURBAN**	87/1.211 7.2	65/810 8.0	51/518 9.8	33/258 12.8	19/121 15.7	255/2.918 8.7	8.7	1.50 (1.26 – 1.79)
**4. RURAL**	18/576 3.1	13/348 3.7	12/277 4.3	9/146 6.2	1/46 2.2	53/1.393 3.8	3.8	0.64 (0.48 – 0.86)

* pop. : Population **P (%): Prevalence

1. URBAN : Populations: Zaragoza, Lista, Pamplona. Studies: ZARADEMP, NEDICES, Pamplona, respectively

2. URBAN MIXED: Population: Hondarribia-Irún. Study: BIDASOA.

3. SUBURBAN: Populations: El Prat de Llobregat, Las Margaritas. Studies: PRATICON, NEDICES, respectively

4. RURAL: Population: Arévalo. Study: NEDICES.

**Table 5:** Comparisons with European data. OR and 95% CI from models controlled for age and sex. Reference: SPAIN

**STUDY**	**AGE INTERVAL (years)**	**POPULATION Denominator**	**Number of cases**	**OR with 95%CI**
**MODEL 1**				
SPAIN *	**75 and over**	6.997	528	**1**
KUNGSHOLMEN (Sweden)	75 and over	984	31	0.40 (0.27 – 0.58)
ROTTERDAM (The Netherlands)	75 and over	2.347	158	0.93 (0.77 – 1.12)
VECCHIANO (Italy)	75 and over	1.136	96	1.16 (0.92 – 1.46)
**MODEL 2**				
SPAIN*	**70 – 85**	8.415	493	**1**
ILSA (Italy)	70 – 85	3.343	280	1.39 (1.18 – 1.64)
**MODEL 3**				
SPAIN*	**70 and over**	10.647	715	**1**
SNES (Italy)	70 and over	2.094	115	0.87 (0.70 – 1.06)

* seven pooled populations
